# Towards a Personality AI for Robots: Potential Colony Capacity of a Goal-Shaped Generative Personality Model When Used for Expressing Personalities *via* Non-Verbal Behaviour of Humanoid Robots

**DOI:** 10.3389/frobt.2022.728776

**Published:** 2022-05-11

**Authors:** Liangyi Luo, Kohei Ogawa, Graham Peebles, Hiroshi Ishiguro

**Affiliations:** ^1^ Intelligent Robotics Laboratory, Department of Systems Innovation, Graduate School of Engineering Science, Osaka University, Toyonaka, Japan; ^2^ Intelligent System Laboratory, Graduate School of Engineering, Nagoya University, Nagoya, Japan

**Keywords:** generative personality models, goal-based personality models, personality AI for robots, robot personalities engineering, humanoid robots

## Abstract

Engineering robot personalities is a challenge of multiple folds. Every robot that interacts with humans is an individual physical presence that may require their own personality. Thus, robot personalities engineers face a problem that is the reverse of that of personality psychologists: robot personalities engineers need to make batches of identical robots into individual personalities, as oppose to formulating comprehensive yet parsimonious descriptions of individual personalities that already exist. The robot personality research so far has been fruitful in demonstrating the positive effects of robot personality but unfruitful in insights into how robot personalities can be engineered in significant quantities. To engineer robot personalities for mass-produced robots we need a generative personality model with a structure to encode a robot’s individual characteristics as personality traits and generate behaviour with inter- and intra-individual differences that reflect those characteristics. We propose a generative personality model shaped by goals as part of a personality AI for robots towards which we have been working, and we conducted tests to investigate how many individual personalities the model can practically support when it is used for expressing personalities *via* non-verbal behaviour on the heads of humanoid robots.

## 1 Introduction

Robots have been part of our lives for a long time. Most robots people know about are characters in fiction: efficient, precise, stalwart, and ever true to their missions. They are already beyond capable in the minds of many who care about them. Fictional robots thus had a head start in defining robots for many. Even “robotics” is a term out of fiction (Asimov, 1941). On the other hand, real robots are far inferior: their limited intellect, their meagre battery life, their awkward, slow movements, and unsteady gait. Their gears grate and motors whine; they may say something silly, awkward, blunt, inconsiderate, or even hurtful; and they do not understand what they are saying or doing. Seldom has a gap between fiction and reality been so vast and striking that it may belie all the hard work of the researchers. Limitations of robots proceed from limitations in the current technology, which is a reality we can only labour through with the steady progress of research. The day will come when real robots catch up with their fictional counterparts. Before that, we have to accept their limitations.

Humans may accept imperfect robots as they accept their imperfect fellow human beings if those robots have likeable or apposite personalities. Apposite robot personalities can facilitate human-robot collaboration (Andrist et al., 2015; You and Robert, 2018) and attenuate the uncanny valley effect (Paetzel-Prüsmann et al., 2021). Robots’ personalities are what enables them to achieve smooth interaction with humans in the long run, when their functions have been taken for granted and their appearances have become the norm.

Engineering robot personalities is a challenging task. Robot personalities engineers face a problem that is the reverse of that of personality psychologists: batches of identical robots freshly off the production lines are to be made into individual personalities, as oppose to formulating comprehensive yet parsimonious descriptions of individual personalities (humans) that already exist. Robots are not humans, who are born unique; robots for practical applications, at least in the near future, will be mass-produced with physical fungibility like computers and smartphones. Although to make every single robot in the world a personality of their own may be too ambitious a goal—for now—we can at least scale down the problem into making populations of robots within which each robot is a personality of their own. We call such a robot population a *robot colony*. The studies so far mostly investigated effects of personality on robots (Mou et al., 2020; Robert et al., 2020). Insufficient are insights into how robot personalities can be engineered. Our incomplete understanding of human personality is partly to blame. More attributable, however, is the lack of a generative model as comprehensive and parsimonious as personality psychologists’ descriptive models.

Thus, we propose in this paper a generative personality model shaped by goals to enable engineering robot personalities in significant quantities. We implemented a proof-of-concept prototype of the model to express *via* non-verbal behaviour personalities of humanoid robots. We carried out tests to study the model’s effective colony capacity when it was used to generate non-verbal behaviour on the head. The rest of this article is organised as follows. Section 2 promotes the establishment of a robot personality paradigm, which is essential for studies like ours but so far insufficient. Section 3 reviews some current generative personality models. Section 4 first presents the model and then the questions to answer in this work. Section 5 relates the tests conducted to answer the questions. Section 6 concludes the study.

## 2 Towards a Robot Personality Paradigm

For the lack of a robot personality paradigm, which is a prerequisite for carrying out studies such as ours, we provide some considerations below. In this paper, a term in the sentence that formally defines it will be italicised in bold. Italics not in bold are just for emphasis.

### 2.1 Definitions

First of all, this work is a study of a novel field that we call ***robot personalities engineering***, a field dedicated to engineering robots into personalities (the plural is for emphasising that it is always about engineering not one but multiple personalities). We define a ***robot personality*** as a robot exhibiting characteristic patterns of computation and behaviour with inter- and intra-individual differences. By “inter-individual differences” we mean that different robot personalities will compute and behave differently in the same context. By “intra-individual differences” we mean that a robot personality will compute and behave differently in different contexts. Inter- and intra-individual differences are described by personality traits. A ***personality trait***, or simply ***trait***, is a unique characteristic of an individual robot defined by a ***broad trait***, a broader facet of the robot’s personality, and a ***trait level***, a value that marks the level (strength) of the broad trait. A ***prominent trait*** is a trait applicable to and distinctly perceptible by a user group in a certain type of robot in its typical usage scenario. Robot personalities are modelled by a ***generative personality model***, a personality model capable of generating behaviour with inter- and intra-individual differences. Every robot personality is based on a ***personality construct***, a particular build of a generative personality model with a unique set of personality parameters. Every personality construct corresponds to a personality; even if we make a copy of a personality construct, the two copies are one robot personality, not two, unless further development makes them different. ***Trait capacity*** is the number of broad traits (personality dimensions) out of all that are applicable to a type of robot a model can model. A ***robot colony***—in the context of robot personalities engineering—is a population of robot personalities situated in close association. By “situated in close association” we mean that those robots will be observed by the same people for their shared qualities or goals. The “colony” here takes a similar meaning to that in expressions such as “a colony of artists”. It may but does not necessarily indicate spatial proximity. For example, a colony of singer robots, albeit not necessarily working at the same place, may be observed by the same audience, and hence each of them needs to be a personality of their own. The essential feature of a colony is that there is no duplication of personalities: each one in a colony is unique. A robot colony should be distinguished from a ***robot population***, which is simply a group of robots with or without personalities. ***Colony size capacity***, or ***colony capacity*** for short, is the number of individual personalities a generative personality model supports. A ***personality archetype***, or simply ***archetype***, is the “target” personality a robot is engineered to be. It is a blueprint or example for a personality construct. An archetype can be represented by a vector in a ***personality space***, which is the space of all possible configurations of personalities defined by a set of personality dimensions, corresponding to several broad traits. The proximity between a robot personality and the corresponding archetype in the personality space is measured by ***fidelity***: high fidelity means high proximity. A robot expresses personality *via* a ***mode of expression***, or simply a ***mode*** ^1^, which is a distinct means of expressing personality associated with a group of actuators^2^ or signalling apparatus. An ***observer*** is an agent that can perform measurements of a robot’s personality. The most common observers are humans. When a significant number of observers reported their impressions on a robot personality using applicable personality assessment scales, the variation among their impressions is measured by ***consistency***: the more varied the impressions are the lower the consistency.

### 2.2 Generative Personality Models

A generative personality model, as the term suggests, is capable of generating behaviour with inter- and intra-individual differences, as opposed to a descriptive model that only describes inter- and intra-individual differences in behaviour. Although we can expect some structural homogeneity between the two breeds, to construct generative personality models we need some understanding of how inter- and intra-individual differences between personalities lead to inter- and intra-individual differences in behaviour in the same situation, which is yet to be fully explored in personality psychology (Baumert et al., 2017). Nevertheless, roboticists’ solutions do not have to be completely faithful to human conditions. Whatever works on robots may suffice.

We postulate that a generative personality model has these key elements: a personality structure that encodes inter- and intra-individual differences in personalities as existing descriptive models do, components that generate behaviour according to situations following some general behavioural tendencies corresponding to certain broad traits, and components that modulate the generated behaviour to reflect the inter- and intra-individual differences.

### 2.3 Model Evaluation

In robot personalities engineering, we consider four criteria for model evaluation: trait capacity, colony capacity, fidelity, and consistency. *Fidelity* and *consistency*, as previously defined, are criteria for evaluating how accurately and consistently a generative personality model can present personality archetypes. They can also be used to measure the quality of engineering of a robot personality (construct). We have explored the details and their usage in our previous study (Luo et al., 2022). In this study, we are more concerned with the other two. *Trait capacity* is the number of broad traits (personality dimensions) out of all that are applicable to a type of robot a model can model. For example, Völkel et al. (2020) have identified 10 personality dimensions—corresponding to 10 broad traits—applicable to speech-based conversational agents. If a generative personality model for the said agents can generate behaviour for 7 of them, the trait capacity is 7 out of 10 or 70 per cent. Usually, we can expand the capacity of a model as long as we understand the behavioural tendencies described by a broad trait and can implement them. *Colony capacity* is the number of individual personalities a model supports. It is the main focus of this study since the colony capacity of a model is a direct indicator of whether it can enable us to engineer mass-produced, physically identical robots into robot personalities of any significant quantities. In theory, any models that support continuous trait levels have infinite capacity. In reality, the effective capacity may be much lower for a number of reasons such as design constraints, hardware limitations of robots, and the complexity of personality perception (Asch, 1946).

### 2.4 Personality Assessment

So far, the most commonly adopted yet at the same time problematic (descriptive) personality model for robot personalities assessment is indubitably the five-factor model (Norman 1963; Tupes and Christal, 1992), alias the “Big Five” (Goldberg, 1990), of which there are many versions (Goldberg, 1993). The arguably most popular version consists of five personality dimensions (broad traits): extraversion, agreeableness, conscientiousness, neuroticism, and openness (McCrae and Costa, 1985). However, little justification there is for that a model originally formulated for humans applies to artificial agents such as robots (Luo et al., 2022). Human-computer interaction researchers have already noticed this issue and taken the initiative in creating their own models, starting with identifying personality dimensions for speech-based conversational agents (Völkel et al., 2020). With all the above said, the five-factor model should apply to a special type of robots, androids, since they are engineered to be as human-like as possible—as well as animation-style (“cartoonish”) androids (Yang et al., 2020), which we call “anidroids,” and virtual humans (Ruhland et al., 2015).

A critical issue at the moment is that there is not a descriptive model dedicated to assessing robot personalities. And it is hard to conceive a model applicable to all robots, of different sizes, shapes, and purposes. What is a model that can be applied to both the rovers on Mars and the animatronics in theme parks? If there really is such a model, it is unlikely to be as parsimonious as the five-factor model. Nevertheless, the five-factor model remains the first choice for many—for assessing social robots at least.

However, neither the lack of a dedicated alternative nor the popularity of the five-factor model justifies its applicability. Before we acquire a dedicated model as Völkel et al. (2020) did for speech-based conversational agents, we need to use the five-factor model with caution. The most basic caution is to check the applicability of the five dimensions, especially for engineering tasks. This is covered by our previous study (Luo et al., 2022).

## 3 Overview of the Current Generative Personality Models

The current generative personality models can be placed into two overlapping categories: goal-based models and models applied to robots. Goal-based models, which, depending on the design, can also be called goal-oriented models, goal-directed models, goal-conditioned models, and so on. There are only a few goal-based generative personality models in robotics research; most are found in virtual agents or narrative generation studies.

### 3.1 Goal-Based Models

Carbonell (1980), in their pursuit of AI that understands stories, proposed a goal-oriented personality model for understanding characters’ personalities and thereby predicting and interpreting their actions. The philosophy behind their “goal-tree” model is that a character’s personality is reflected in their actions in a situation, and those actions point ultimately to their goals, thereby associating their goals with personality. Their model is not generative *per se* but can be easily adapted into one, since predicting the actions is in a sense generating hypothetical actions. To create virtual characters with inter-individual differences and autonomy in behaviour, Chittaro and Serra (2004) proposed a goal-oriented model, where personality traits influence how characters sense the world, plan their actions, and how the actions are performed. The goals are represented by probabilistic finite-state machines, where the trait levels are used as weights to compute probabilities of state transitions. However, the model requires for each goal a probabilistic finite-state machine, which is easier to define in a virtual, closed world. Yet in a real, open world, the means to achieve certain goals are often unclear and hence unable to guide the behaviour or choices of actions. Bouchet and Sansonnet (2011) proposed a model for implementing influences of personality traits on cognitive agents. The influences are defined by associating to personality traits sets of “bipolar schemes,” which consist of eight classes of “level of activation” operators that can be applied to multiple phases, from goal selection to the execution of a plan for achieving the goal to the evaluation of what has been done, of the five-phase deliberation cycle of the *BDI-agent* (Rao and Georgeff, 1995). The authors then had not yet done empirical assessments of their model. Bahamon and Young (2017) proposed a narrative generation system, *Mask,* featuring an algorithm, *CB-Glaive,* based on the work of Ware and Young (2014). Their *Mask* system can choose for a character a course of action that aligns best with their goal and hence continue the narrative from some given premises with available choices of actions.

Many goal-based models are considered in narrative generation or analysis studies. The above mentioned study by Carbonell (1980) is one of the seminal contributions to narrative analysis. However, most current robots cannot deliberate goals, plans, or decisions on courses of actions for actionable, real-world behaviour. There are not many attempts on goal-based approaches to “high-level” behaviour. Still, the flourishing field of machine learning is filling the gap. Goal-based or goal-directed reinforcement learning has striking parallels in narrative generation or other artificial agents studies. A major difference is that goals as defined in reinforcement learning usually have nothing to do with personality.

### 3.2 Generative Personality Models Applied to Robots

So far, the most common type of generative personality models applied to robots are “specialised” models, which are specialised to generate in very specific situations very specific aspects of behaviour associated with a few personality traits. There is the gaze model by Fukayama et al. (2002) for virtual agents, which Luo et al. (2019) showed to be applicable to androids with necessary modifications. Lee et al. (2006) implemented their system on *AIBO*, a robot dog. The system had two sets of hard-coded hardware configurations corresponding to binary personalities, extroverted and introverted, on the extraversion dimension. Such an approach is applicable to companion robots or social robots that have no needs to form large colonies. For humanoid robots that work as humans’ co-workers or helpers in various domains where multiple robots will work and hence be observed together, a generative personality model of a large colony capacity is required. Aly and Tapus (2013) devised a model capable of generating verbal and non-verbal behaviour for robots. It works by generating personality-modulated speech and then personality-modulated gestures out of the personality-modulated speech. For their experiment, the authors tested two personality configurations on the extraversion dimension. Their model lacked a structure to encode inter- and intra-individual differences for more than two personalities on more than one dimension. It is unclear whether their model has the potential to express more personalities *via* possible expansions. The personality-driven body motion synthesis models by Durupinar et al. (2016) contributed an insight about “effort” into personality. The gist of their method is to map personality traits to “Effort,” as defined in Laban Movement Analysis, and then “Effort” to body motion parameters. Many human activities involve “effort”—albeit defined differently in different contexts—and therefore the idea may apply to a range of human behaviour other than body motion. Zabala et al. (2021) proposed an affect-based system that generates non-verbal behaviour consistent with the robot’s speech. Their system does not have components to encode inter- and intra-individual differences of personalities by traits but works by generating non-verbal behaviour from the emotions expressed in the robot’s speech. Speech is an important means of personality expression, and therefore their system has the potential to be integrated into a generative personality model for speech. Better understanding of personality traits and inter- and intra-individual differences in emotional expressions *via* speech may be necessary to achieve the integration. Churamani et al. (2022) proposed a framework for constructing models capable of generating affect-modulated behaviour based on the appraisal of users’ affective states on the arousal and valence dimensions, which is in a sense generating behaviour with intra-individual differences, considering that users’ affective states are one of the most important contexts, among a few others, in expressing or understanding robot personalities. And by configuring it to make the robot behave either patiently or impatiently, or to be either excitatory or inhibitory in generating the robot’s mood, the model can generate behaviour with inter-individual differences for at least two distinct individuals on each of the three broad traits (being generous, persistent, and altruistic) that the authors confirmed to be consequential in the kind of human-robot collaboration they studied. The authors did not advertise their framework to be meant for constructing generative personality models *per se*, but the potential is clear from their results. Only that personality encompasses more than just affective behaviour. How to encompass other aspects of personality and how to use their framework to construct models capable of generating behaviour with inter- and intra-individual differences for a significant quantity of robots were not the aims of their published works and hence unknown.

It could be hard to integrate those specialised models without compromising the overall consistency of personality, an essential quality of personality (Isbister and Nass, 2000). In other words, it will not be easy to put specialised models together to create a robot personality with a consistent overall impression. Another common drawback is that most of those models present broad traits in very low resolution; most of them support two discrete trait levels (high, low). Being specialised models with also discrete (often binary) trait levels severely limits the colony capacity of those models. Furthermore, many of the specialised models for robots, such as the eye movement model applied to androids (Luo et al., 2019), have no structure to encode inter- and intra-individual differences of personality, and hence the links between personality structures and behaviour remain obscure. Such models have limited practical value for engineering robot personalities in significant quantities.

## 4 Proposed Model and Research Questions on Its Capacity

Similar to the previous works, such as that of Chittaro and Serra (2004), the proposed model uses personality trait levels as probabilistic influences over behaviour. The main difference is that the proposed model does not require associations between behaviour and goals and is thus more applicable to robots in the real world. As in reality, one often does not know for certain whether some behaviour is conducive or leads to the attainment of a goal. (Does studying hard in school lead to success? Or is it that successful people tend to work harder than average as one of their traits?) The proposed model associates behaviour with personality traits and then personality traits with goals. Instead of “goal-oriented,” we describe the proposed model as “goal-shaped”. The philosophy is that the robot’s personality structure can be shaped to facilitate the attainment of a goal even when there is no reliable knowledge on whether certain behaviour helps achieve the goal. However, the objective we set out to achieve here is to enable engineering robot personalities in significant quantities out of mass-produced, physically identical robots. How does our design philosophy serve this objective? For the answer, we propose this working principle: *the root of all inter- and intra-individual differences in computation and behaviour is different goals*. Different personalities behave differently in the same context because their goals are different. A personality behaves differently in different contexts because their goals are different in different contexts. The goal-shaped structure of the proposed model is how it generates behaviour with inter- and intra-individual differences. We might be more interested in inter-individual differences for the capacity is crucial for robots employed in significant numbers. Imagine someone going to work. They see one of their robot errand runners and say hello. But a few steps further, they bump into another, looking the same as the one before and offering greetings in the exact same manner, and then another, followed by another, and again, and again, and again, and one cannot get to their cubicle until this has happened a dozen times (everyday). The image of those robots is that of many copies of the same machine; it would not make them likeable and easier to forgive as we have hoped. The interaction they offer is grimly tedious, reminiscent of a working environment where everyone is but a replaceable cog in the machine with no individuality. It would be significantly more interesting if the errand runners possess individual personalities, formed and shaped in the course of them working alongside their human colleagues, and offer characteristic interaction showing their individuality.

### 4.1 Personality AI for Robots and the Proposed Personality Model

**FIGURE 1:**
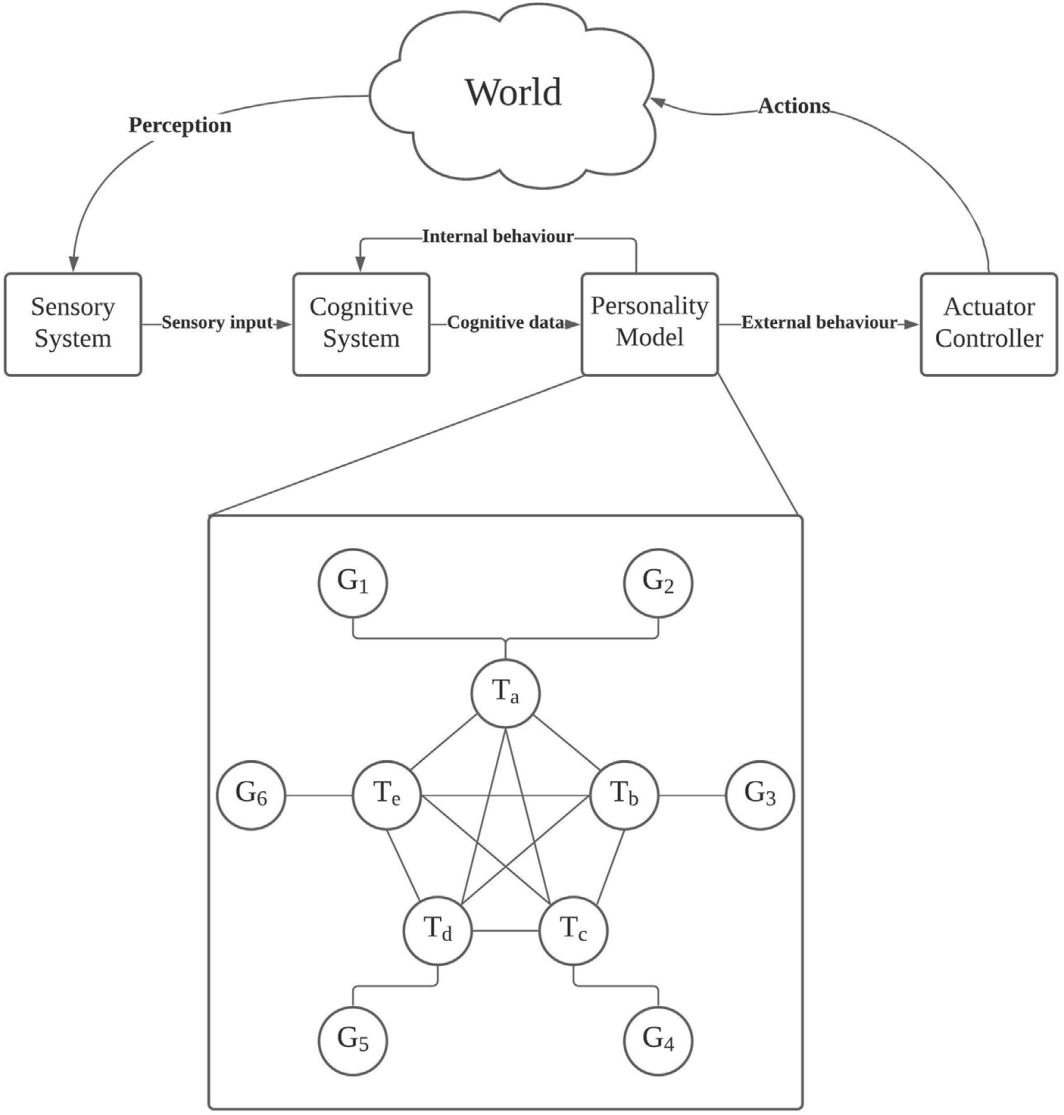
A diagram for an example model.

Consider the robot AI shown in Figure 1, towards which we have been working. It is, generally speaking, of the “Sense-Plan-Act” paradigm but with a personality model^3^ in the “Plan” part. It consists of a sensory system to perceive the world to produce sensory output, which a cognitive system takes as its input for cognitive output, such as understanding of situations, which the personality model takes as the input to generate behaviour, which includes external behaviour (movements, actions, social behaviour, and anything observable) and internal behaviour (predispositions, moods, emotions, and anything not directly observable). Indeed, the “behaviour” here refers not only to specific instances of actions but a range of possibilities, from the activation of a process to running a long-term scheme. Saying hello is behaviour. Committing oneself to pursue a goal over a long period of time is also behaviour. Everything an AI can do can be behaviour and can be influenced by its personality. Finally, the actuator controller interprets external behaviour into actuator commands, whereas the internal behaviour acts back upon the personality and cognition. This particular study does not aim to cover every part of the personality AI but focuses on the personality model.

The personality model consists of a graph **Γ** of nodes **V** and edges **E**. Formally,

$$\Gamma =\left(V,E\right).$$ (1)

There are two types of nodes: goal nodes **G** and trait nodes **T**:

V=G∪T. (2)

A goal node represents a ***goal***, which is the objective the robot is operating to achieve, and hence a goal may also be called an *objective*, as in “objective function,” or a *directive*, as often so called in fiction. The exact implementation of a goal node depends on the type of robot and its purposes or typical usage. Generally speaking, a goal node should report a measurable difference between the *actual state* and *goal state*. For instance, if a personal trainer robot is tasked to make sure that their client exercises for 30 min a day, everyday the goal node output would start at 30 min, that is the difference between the actual state, 0 min, and the goal state, 30 min. When the robot has nudged their client into exercising, the goal node output starts to decrease, and when they have exercised for 16 min, the goal node should report 14 min as the difference between the actual state and goal state. When the difference becomes zero, the goal is achieved for the day. Certainly, not all goals admit measurements that can be so simply defined. The goal nodes may be arranged into a structure, such as a tree (Carbonell, 1980), to encode priority (not shown in Figure 1) and thus resolve conflicts between goals if the robot has multiple goals. Structures of goals are beyond the scope of this work.

The trait nodes form a clique. A trait node represents a broad trait. The purpose of the node is to use the corresponding trait level (as a behavioural tendency parameter) and cognitive input to generate behaviour as the output. The cognitive input consists mainly of cognitive states; a ***cognitive state*** is an understanding of the situation, which can be a belief, thought, or any other simulated psychological state. We consider such an implementation of a trait node: it consists of a ***mapping function*** that maps cognitive states to behaviour:

$$f_{m}:S\to B,$$ (3)

where S denotes the space of cognitive states, and B , the space of behaviour. And a probabilistic ***commitment function*** that, as the name suggests, generates *commitment* to the possible behaviour from the mapping function. Given a cognitive state, the trait node, using the mapping function, first generates an ***urge*** for certain behaviour, which is an instance of behaviour not yet put into action, with a probability scaled from the trait level. For example, 0.629 is the probability of generating an urge by a trait node with a trait level of 4.4 out of 7. In this study, we consider trait levels on a range of [1, 100] for convenience. For behaviour of which the tendency is the complement of that suggested by the trait level, such as introverted behaviour managed by an extraversion trait node—the probability to generate an urge is computed from the complement of the corresponding probabilistic tendency of extraversion. For instance, urges of introverted behaviour are generated at a probability of 0.3 when the extraversion trait level is 70 out of 100. The mapping function having generated an urge, the commitment function then computes the probability of achieving or not achieving the current goal given event *B*:

$$P\left(G\cap B\right)=P\left(G|B\right)P\left(B\right)$$ (4)

and

$$P\left(G^{\prime }\cap B\right)=P\left(G^{\prime }|B\right)P\left(B\right),$$ (5)

where *G* denotes the attainment of the goal, that is the goal node reporting no difference between the actual state and goal state, *B* denotes putting the urge into action, and *P* (*G*|*B*) and *P* (*G*′|*B*) are conditional probabilities provided by the cognitive system based on its understanding of how the world works. When there is not sufficient understanding to acquire *P* (*G*|*B*) and *P* (*G*′|*B*), the model assumes the two to be equal: *P* (*G*|*B*) = *P* (*G*′|*B*) = 0.5. If *P* (*G*′ ∩ *B*) is bigger than *P* (*G* ∩ *B*), indicating that the behaviour will work against the goal, the urge is *suppressed*. It is entirely possible that to do nothing might serve the goal best, that is when *P* (*G* ∩ *B*′) is higher than both *P* (*G* ∩ *B*) and *P* (*G*′ ∩ *B*), thereby making the commitment a less than optimal choice either way. We might still want personality traits to function based on action rather than inaction since robots are designed to do things and probability is not certainty. We call a personality with trait nodes functioning based on action a ***proactive personality***. (That said, the possibility is there to implement a non-proactive personality should the circumstances demand it.) Consider that, as with humans, oftentimes, a personality trait does not contribute to a goal in the best way possible—if it does not work against the goal. We might still want to leave it be as it may contribute well to another goal.

If the reader wonders how the model handles certainty of actions required from the robot, such as following orders and sticking to daily routines, the behaviour we consider here is not necessarily specific actions; it can be a state that guarantees certain actions. When such a state, say sticking to daily routines, is switched on by the personality model, the robot will stick to daily routines without fail, until changes of circumstances trigger another state as a new behavioural pattern that requires the robot to break out of their daily routines. The same may apply to following orders. When robots committed themselves by their personalities to following orders, they will follow all orders directed at them until the model determines that it is no longer necessary.

A personality trait may not contribute to a goal in the best way possible, and by the following design, this trait may not be in a prioritised position to contribute. For each goal of a robot, there exists an ***order*** of trait nodes arranged in accordance to their contribution to the goal: a trait node that contributes more to the goal will be ranked higher in the order. There can be many approaches to acquiring an order. Here we proffer two heuristics. **Heuristic A:** If a robot can develop their trait levels to facilitate the attainment of a goal or is thus developed, a way to acquire an order is to sort trait nodes by trait levels from high to low, since that, if a trait node contributes more to a goal, the corresponding trait level may in time grow higher or be made so in the first place. However, this is not always the case unless the goal is the most important to the robot (their mission), and there might be cases where being low on a personality dimension contributes most to a goal but the corresponding trait node will never be ranked high by the heuristic. This heuristic should be used with caution. As a rule of thumb, we recommend using Heuristic A only when there is no better choice. Another heuristic is to assign scores to all trait nodes. **Heuristic B:** Beginning with equal scores, say zeros, and whenever the behaviour triggered by a trait node has reduced the goal node output, the score of the corresponding trait node shall be increased. The trait nodes will be sorted by the “contribution” scores according to preset time intervals to acquire or update the corresponding order.

Robots who have similar goals but in different environments may have different trait orders. For example, neuroticism—a trait that relates to avoidance motivation (Elliot and Thrash, 2002), as to avoid undesirable possibilities or results—of a rover on Mars may be ranked higher than a rover on the Moon since a Mars rover may need to avoid dust storms, whereas a Moon rover faces no such danger. The trait of the highest-ranking trait node concerning a goal is the ***primary trait*** for the goal. A robot’s most important goal of their existence is the robot’s ***mission***; the primary trait for the robot’s mission is the robot’s ***dominant trait***. It can be expected that robots in the same colony have the same mission, since it is the purpose of those robots’ existence and shall exist over a long time if not to last throughout the robots’ service lifespan. A robot works towards their mission through achieving a series of short-term, concrete goals just as humans do, and those short-term goals may differ according to the environments and circumstances the robot finds themselves in. Thus, there exists a causality gap between the robot’s concrete behaviour and mission. This gap can be helpful since some behaviour that seems inconsequential or even detrimental to the mission may be necessary to a short-term goal that is essential to the mission. Short-term goals may be broken down into shorter-term goals and the same situation may persist. This is why it is important to dissociate behaviour with goals and reconnect the two with personality.

**FIGURE 2:**
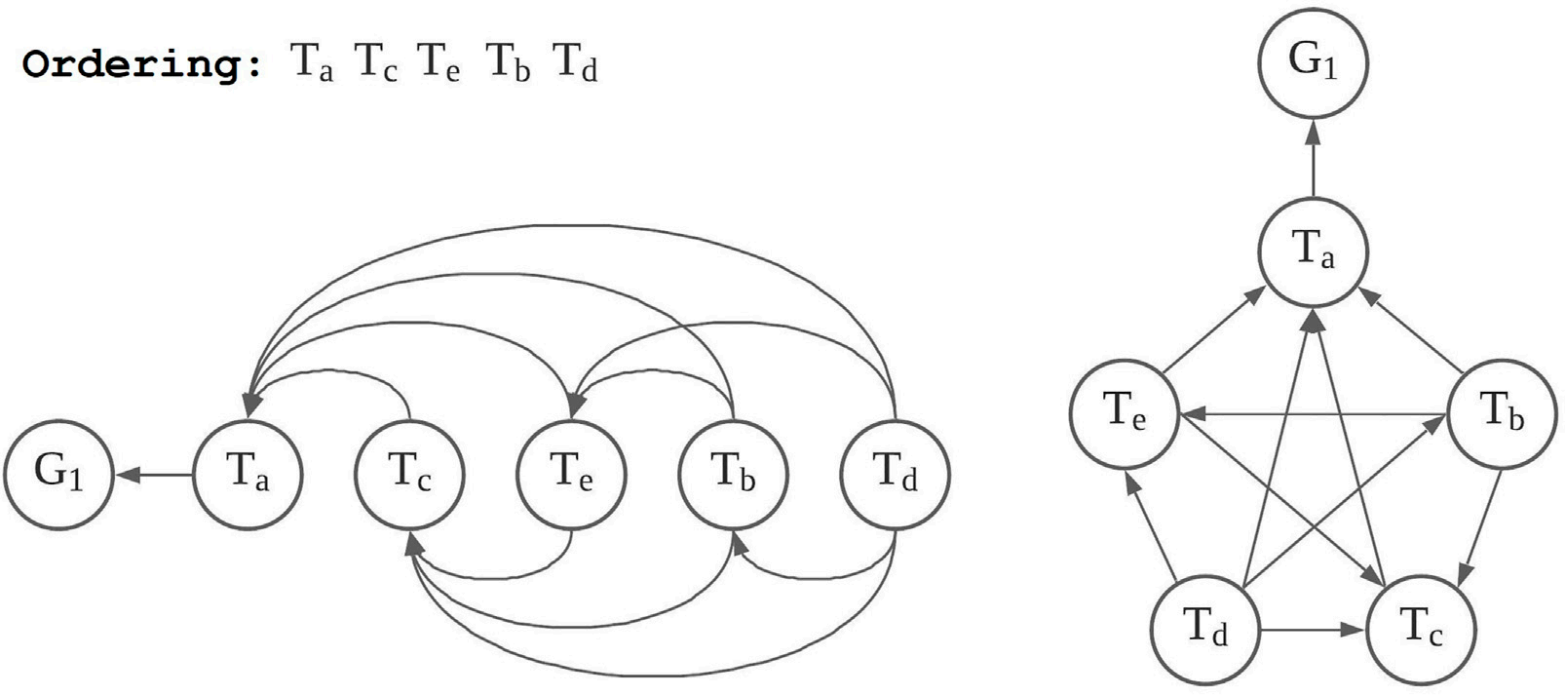
An example of a goal-active form of the example model.

Given a goal, which demands an order of traits, we can transform the personality model into the corresponding ***goal-active form*** (Figure 2); the transformation is thus done: we look only at the goal towards which the robot is currently working, namely the robot’s ***active goal***, and the clique of trait nodes, then we convert the graph into a directed one by designating directions to the edges following such a rule: for every pair of trait nodes, the orientation of the edge between them is always from the node lower in the order to the node higher, and the edge from the node highest in the order points to the goal node.

When the robot is working towards a certain goal, the personality model of the robot is in the corresponding goal-active form. For the time being, we do not consider a robot capable of working concurrently towards multiple goals. The robot works towards one goal at a time, and if they need to work towards multiple goals, their personality model has to switch between the corresponding goal-active forms as how a CPU core handles multitasking. How a robot switches between goals, the interruption mechanisms and such, is beyond the scope of this work. We only assume that the robot’s mission stays active by default when there is not another goal being active. The goal-active form corresponding to the robot’s mission is the default goal-active form of the robot’s personality construct. When a robot personality model is in a goal-active form, it generates behaviour out of a cognitive state following these rules, from the highest trait node (the one connected to the goal node) in the order to the lowest one by one, check against all of the connected lower nodes, and if 1) the behaviour required by a lower node is in conflict with that of the current node, the urge for the behaviour is nullified, and the lower node is marked as “to be skipped”; 2) the same, the behaviour is enhanced, and the lower node is marked as “to be skipped”; 3) neither in conflict with nor the same as the current node, do both; 4) skip all nodes previously marked as “to be skipped”. Here, by “in conflict with” we mean that only the behaviour of one of the trait nodes can be put into action. By “enhanced” we mean “done with more effort,” which can be interpreted according to the behaviour. For example, a smile can be enhanced into a grin; working can be enhanced into “working hard” and further into “working harder”. We can implement the rules and designs stated above into algorithms. Algorithm 1 is an example. The details can vary, such as the handling of *effort*, which need not be discrete levels enhanced by increments.

Algorithm 1 An Algorithm to Generate Behaviour

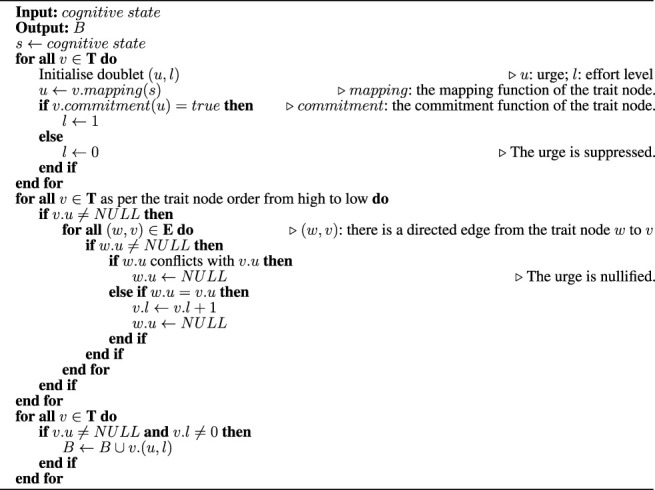

If the reader wonders how a personality model that changes into different goal-active forms can ensure the consistency of personality expressions, it does not—to the fullest extent. As in real life with humans, a person who is engaged in a goal may “seem like a different person”. For instance, a person who is introverted but works as a receptionist may be constantly offering warm smiles and promptly engaging customers as if they are an extrovert. Oftentimes, a person may simply act “out of character” in certain situations. If a robot—when in a goal-active form pertaining not to their mission—exhibits an impression of personality different from that of their true personality, we call this impression of personality their ***persona***. Normally, the true impression of personality is exhibited when the robot is in the goal-active form of their mission.

The model proposed here is compatible with personality development processes. Robots with apposite personality development subroutines can foster traits that contribute to their goals by adjusting the trait levels. Personality development is an essential topic in personality psychology (Baumert et al., 2017). The development of a subroutine may need the corresponding advancement in personality psychology.

The model is for robots and therefore not necessarily a faithful representation of human personality. The separation of cognition from the personality model is likely not true with humans. In robot personalities engineering at least, it may benefit to engineer the robot’s cognition as a separate system that may or may not be influenced by their personality.

### 4.2 Research Questions

Having formulated the model, the first question to explore is *what is the colony capacity of the model?* We are interested in the colony capacity first when assessing any generative personality models because the criterion is the direct indicator of whether the model can be used to engineer a large number of robots. The capacity of the proposed model, in theory, is infinite since the model uses continuous trait levels as behavioural tendency parameters.

In reality, however, there are constraints and limitations. Since we aim to engineer likeable robot personalities, low conscientiousness and low agreeableness configurations are out of consideration. Moreover, if we use the model for personality expressions, as we are motivated to do, there is no guarantee that the users can tell the subtle differences between two similar personalities. Therefore, we conducted tests to answer such a question: *what is the effective colony capacity of the proposed model when it is used to express the personalities of humanoid robots via the non-verbal behaviour on their heads?*

### 4.3 On the Scope of the Questions

Considering our initial motivation, that is making imperfect robots more likeable, one of the first applications of the proposed model is none other than expressing likeable personalities. But why should we focus on humanoid robots and their heads? Humanoid robots should be commonplace in human environments, and they are the robots who need to interact with humans. They are expected to express their personalities mainly *via* non-verbal behaviour because their speech patterns may be controlled or restricted for consistent user experience and minimising issues such as offending the users or saying something controversial until natural language processing has advanced far enough to handle characteristic yet user-friendly speech. Of all non-verbal modes, those on the head should be the most common and safest. Human-like gestures may be an option for virtual agents, whereas robots swinging their steel arms about can be a safety hazard for the humans nearby. Limiting the non-verbal behaviour to the head well reflects the limitations early robots may be subject to when expressing their personalities.

## 5 Tests

### 5.1 Overview

Two tests were conducted to study the effective colony capacity of the model in the given settings. In each test, 30 observers assessed the personalities of the robots based on the head, eye, and smiling behaviour they exhibited in 8 94- or 95-s long videos^4^. The cover story featured in the videos was that the robots were watching and listening to another robot of the same model conversing with a human:

HUMAN: Are you SK … ?

ROBOT: I’m SK-0.

HUMAN: SK-0, OK. ... Frankly, it’s almost impossible to tell you guys apart just by the looks. But I suppose the same could be said for most non-human animals, such as the rats in the lab.

ROBOT: You could acquire the capacity over time.

HUMAN: Do all humans look the same to you?

ROBOT: No, they don’t.

HUMAN: What about animals?

ROBOT: It depends.

HUMAN: Um … could you tell two pigs of the same breed and size apart?

ROBOT: Yes, I could. No two pigs are alike.

HUMAN: Do you think that you could tell aliens apart?

ROBOT: I don’t know. It would depend on their features.

HUMAN: Do you think that humans could tell aliens apart?

ROBOT: If they have trouble telling animals apart, then it would probably be the same for aliens.

HUMAN: That might be a problem. Maybe Earth’s first ambassador to an alien civilisation should be a robot.

ROBOT: Could you elaborate?

HUMAN: If humans wouldn’t be able to tell aliens apart—not confidently anyway—then that would be a big disadvantage in diplomacy.

ROBOT: I see. Perhaps then it would be better to send robots to meet aliens. Suggestion: start developing diplomatic robots now.

HUMAN: Diplomatic robots? Now?

ROBOT: Yes. The first contact could be tomorrow. It could be today.

**FIGURE 3:**
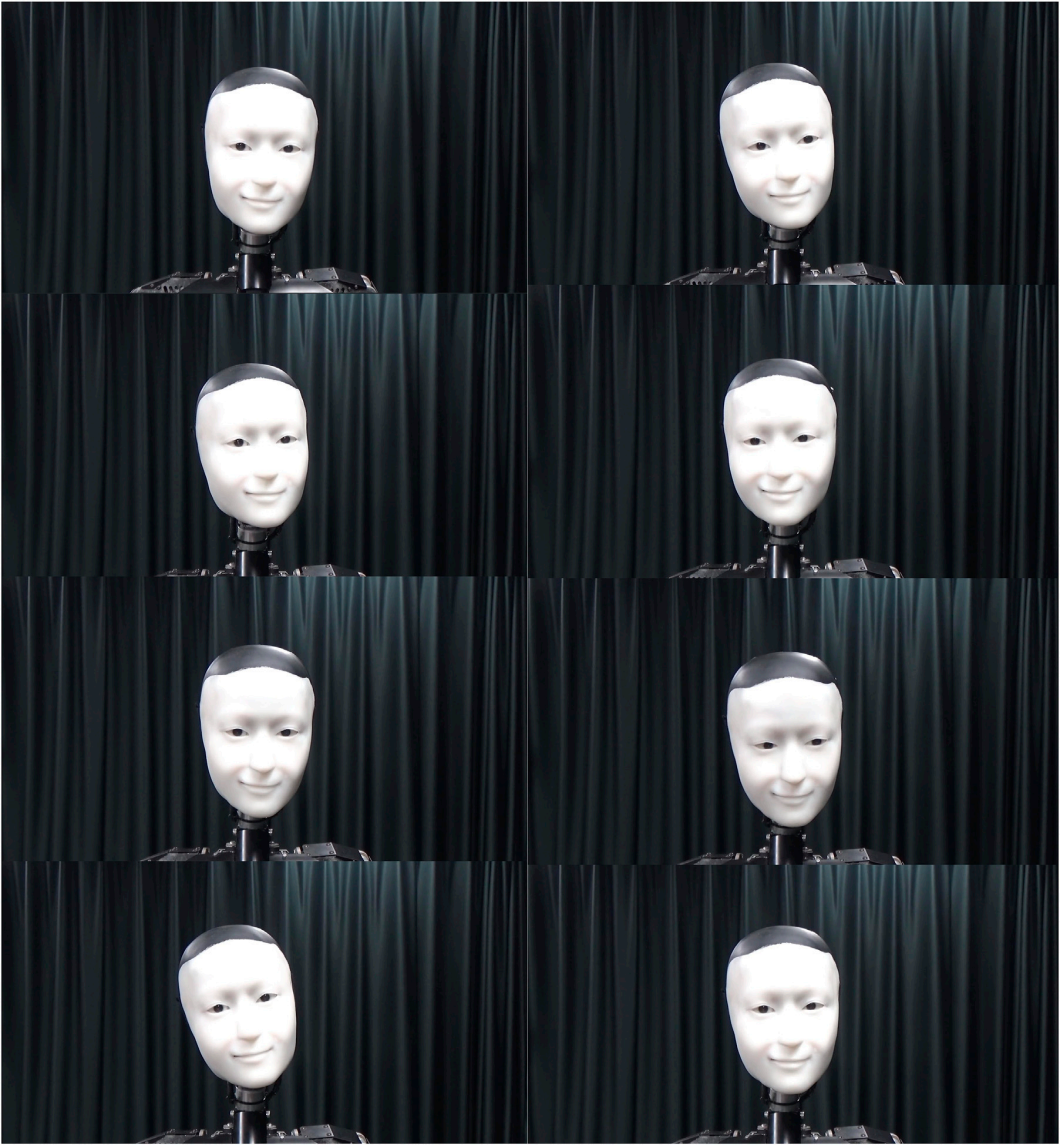
The robot tested (one robot with multiple personalities).

The videos were taken right in front of the auditor robot personalities (Figure 3). All aspects of all short videos were the same except for the robot’s non-verbal behaviour.

To make the videos, we had created 12 robot personality archetypes (Table 1). The extraversion trait levels were thus chosen and arranged to include a range of extroverts and introverts. The agreeableness and conscientiousness trait levels were generated randomly with the constraints that they must be higher than 50 to reflect possible real-world design constraints for service robots. We then constructed 12 personality constructs corresponding to 12 robot personalities as per the designs described in Section 4.1. Test 1 featured Robot Personalities 1 to 4, and Test 2, Robot Personalities 5 to 12. In each test, we created a survey with eight pages requesting the participants first to watch the video on that page and then report their impressions of the robot. Each robot personality in Test 1 made appearances in two videos. The eight videos appeared in random order for counterbalancing. There was also the introductory page introducing the scenario of the cover story, which was described to the participants as below but not shown to them. On the page it said:

**TABLE 1:** The 12 archetypes for engineering 12 robot personalities.

Archetypes (Corresponding Robot Personalities)	Trait Levels^a^			Numbers of Appearances	
	Extraversion	Agreeableness	Conscientiousness	Test 1	Test 2
Archetype 1 (Robot Personality 1)	5	93	57	2	0
Archetype 2 (Robot Personality 2)	25	69	96	2	0
Archetype 3 (Robot Personality 3)	75	91	85	2	0
Archetype 4 (Robot Personality 4)	95	85	67	2	0
Archetype 5 (Robot Personality 5)	10	88	65	0	1
Archetype 6 (Robot Personality 6)	20	78	58	0	1
Archetype 7 (Robot Personality 7)	30	60	88	0	1
Archetype 8 (Robot Personality 8)	40	78	92	0	1
Archetype 9 (Robot Personality 9)	60	77	86	0	1
Archetype 10 (Robot Personality 10)	70	98	90	0	1
Archetype 11 (Robot Personality 11)	80	77	84	0	1
Archetype 12 (Robot Personality 12)	90	59	70	0	1

a The range of trait levels is from 1 to 100.

In a certain robotics laboratory, a robot, SK-0, was engaged in discussion with a human, and SK-0’s fellow robots of the same model, SK-1 to SK-n, were watching and listening to the two of them. Since they were of the same model, their appearances were all identical; their only differences were their personalities. The following 8 videos have recorded their behaviour in the discussion session. There can be multiple videos featuring the same robot personality. Please observe them and report your impressions of them.

Right after the eight personality assessment pages was another page requesting the participants to report how many robot personalities they thought there were in the videos they had watched.

The personality assessment form used was based on the 10-item personality assessment inventory proposed by Rammstedt and John (2007), which is based on the 44-item original version, “the Big Five Inventory,” by John et al. (1991). We made some modifications. First, items on openness and neuroticism were removed since they were not applicable to our robot (Luo et al., 2022). Second, we changed the five-point Likert scales to seven-point by adding “agree” and “disagree”. Correspondingly, the reversed items were computed by subtracting the score from 8. Third, we changed the object of the question from “myself” to the robot personality since we were enquiring about the robot. Fourth, we changed the item “tends to be lazy” to “tends to remain idle” since “lazy” was not an appropriate adjective to describe a robot. Issues of imprecise or impertinent adjectives are prevalent when using personality assessment inventories designed for humans to measure robots, as this violates the lexical hypothesis (Galton, 1884; Goldberg, 1990).

For the data analysis, we first aggregated pair-wise comparisons using Euclidean distances and cosine similarity over the 30 sets of reports to verify if the participants could distinguish any pairs of robot personalities. Then, we compared the distributions of subjects as per the reported numbers of personalities in the two tests.

### 5.2 Cognitive States Annotation

To acquired the cognitive states required for the model to work, we conducted a survey using crowdsourcing (all crowdsourcing service reported in this study was provided by Amazon Mechanical Turk). We let 20 participants select from a drop-down list before each line of ROBOT the word which they thought best described ROBOT when ROBOT said the line. The list contained: *agreeing, disagreeing, interested, intrigued, engaged, indifferent, uncertain, confused, frustrated, annoyed, offended,* and *none of the above*.

If more than half of the participants had selected a word, we would accept that word as our cognitive state for the corresponding line. Otherwise, we would choose from the words that had got votes from the participants the one we thought to be the best. We annotated by ourselves a few “polite nodding points” as well, which were when ROBOT would nod to HUMAN while he was speaking. The reason the cognitive states of ROBOT, a character in the scenario of the cover story, applied to the auditor robots too was because they were all of the same model. The annotated dialogue, with all of the annotations in brackets and bold:

HUMAN: Are you SK … ?

ROBOT: [**Engaged**] I’m SK-0.

HUMAN: SK-0, OK. [**Polite nodding point**] ...Frankly, it’s almost impossible to tell you guys apart just by the looks. [**Polite nodding point**] But I suppose the same could be said for most non-human animals, such as the rats in the lab.

ROBOT: [**Annoyed**] You could acquire the capacity over time.

HUMAN: Do all humans look the same to you?

ROBOT: [**Disagreeing**] No, they don’t.

HUMAN: What about animals?

ROBOT: [**Uncertain**] It depends.

HUMAN: Um … could you tell two pigs of the same breed and size apart?

ROBOT: [**Agreeing**] Yes, I could. No two pigs are alike.

HUMAN: Do you think that you could tell aliens apart?

ROBOT: [**Uncertain**] I don’t know. It would depend on their features.

HUMAN: Do you think that humans could tell aliens apart?

ROBOT: [**Engaged**] If they have trouble telling animals apart, then it would probably be the same for aliens.

HUMAN: That might be a problem. [**Polite nodding point**] Maybe Earth’s first ambassador to an alien civilisation should be a robot.

ROBOT: [**Interested**] Could you elaborate?

HUMAN: If humans wouldn’t be able to tell aliens apart–not confidently anyway [**polite nodding point**]–then that would be a big disadvantage in diplomacy.

ROBOT: [**Engaged**] I see. Perhaps then it would be better to send robots to meet aliens. Suggestion: start developing diplomatic robots now.

HUMAN: Diplomatic robots? Now?

ROBOT: [**Engaged**] Yes. The first contact could be tomorrow. It could be today.

To avoid interference from ROBOT, who according to the survey expressed their own personality through their lines and voice, was why we adopted a scenario where the robot personalities behaved as auditors that did not speak.

After we acquired the cognitive states (as in brackets and bold; their positions in the annotated conversation were when they would be triggered), we produced the videos as the stimuli. An experimenter had played the voice track and timed the cognitive states while the camera was rolling. A prototype model controlled the robot to exhibit different patterns of behaviour at the same cognitive input.

### 5.3 Systems

#### 5.3.1 Details of the Prototype Model

**FIGURE 4:**
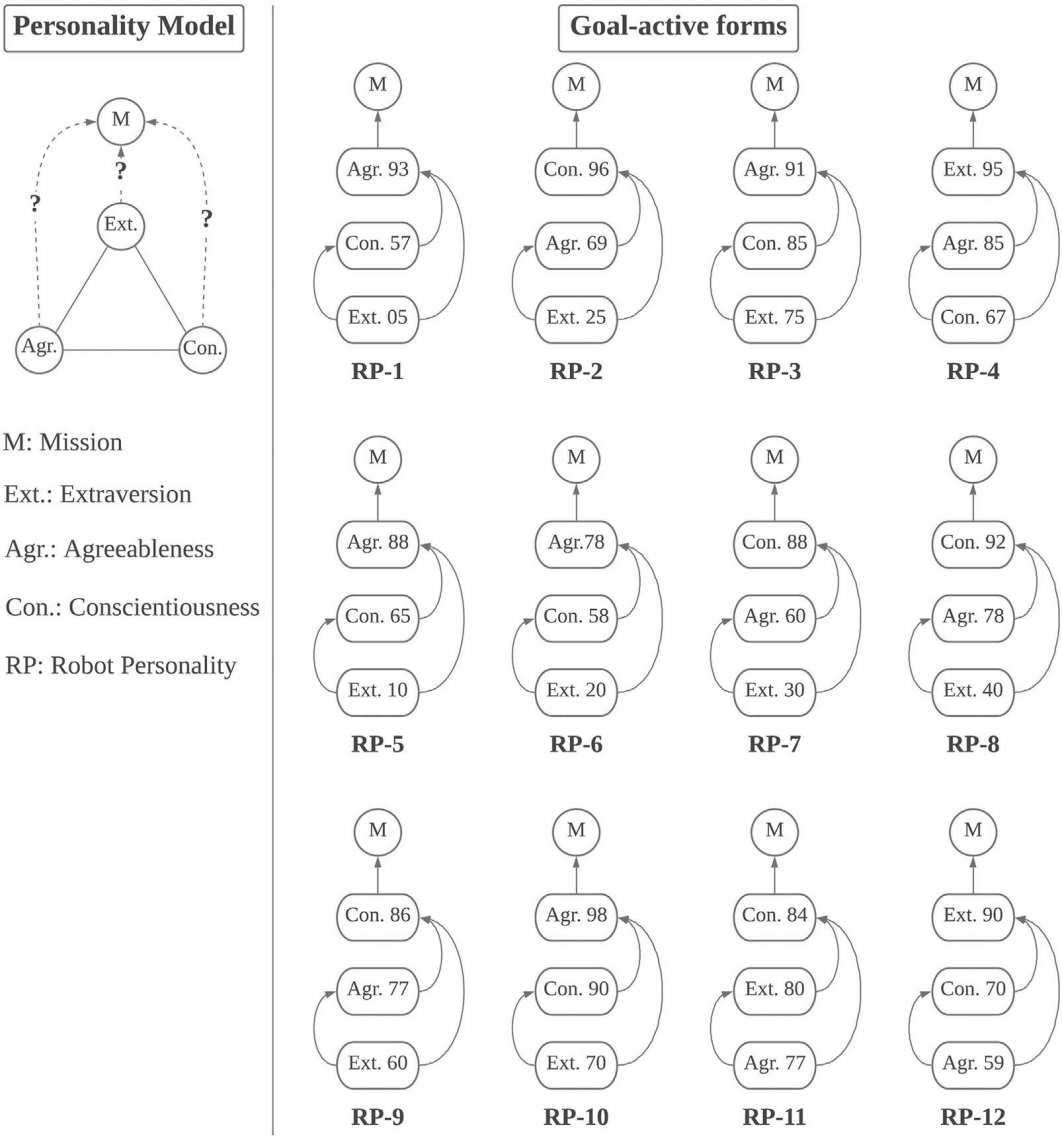
The tested prototype model implemented as 12 personality constructs in their default goal-active forms.

Figure 4 illustrates the generic structure of the prototype model we tested (on the left side) and the 12 personality constructs in their corresponding goal-active forms of their missions (on the right side). The prototype modelled three broad traits of the five-factor model: conscientiousness, agreeableness, and extraversion. We assumed that the robot personalities had shared the same mission but worked in different environments towards different concrete goals, thereby developing different personality traits. We assumed that the active goals of the robot personalities in the scenario were their missions, since they were attending a discussion session featuring a topic that might affect the whole organisation the robots were working for (the scenario of the cover story should be similar to a corporate meeting). In that case, there were no other active goals in effect. We applied Heuristic A and acquired the orders to form their goal-active forms.

The model handled three modes: eye movement, head movement, and facial expression. Table 2 shows the behavioural mappings; Table 3, the details of the behaviour; and Table 4, some predetermined conflicts. The behaviour in Table 3 consisted of pre-recorded actuator sequences, meaning the robot would perform the same behaviour (e.g., a nod) in exactly the same way. Some behaviour was programmed with multiple effort levels, the action of each level was realised by a separate actuator sequence. The contents of the tables should be used according to the rules introduced in Section 4.1 to generate behaviour. Take Robot Personality 10 for example, when HUMAN says “But I suppose the same could be said for most non-human animals, such as the rats in the lab,” the robot is annoyed since their kind is juxtaposed with lab rats. Given the “annoyed” state, the “extraversion” trait node will first generate an urge to roll the robot’s eyes with 0.7 probability, but the “agreeableness” trait node will generate an urge for a (forgiving) nod with 0.98 probability, and the “conscientiousness” trait node will do nothing. Let us assume that both the urge to roll eyes and the urge to nod are generated. Our “dummy” commitment functions will then give in to all urges. According to the rules in Section 4.1 and Table 4, the urge to roll eyes conflicts with that to nod. Since the “extraversion” trait node is lower than the “agreeableness” trait node in the order of the current goal-active form of Robot Personality 10, the urge to roll eyes is nullified and the corresponding trait node will be skipped. The discussion continues, and Robot Personality 10 keeps listening. Now, HUMAN says “Maybe Earth’s first ambassador to an alien civilisation should be a robot,” the robot is interested. According to the behavioural mappings, urges to tilt forward, tilt to the left, and also tilt forward are generated by the “extraversion,” “agreeableness,” and “conscientiousness” trait nodes respectively, assuming chance favours it. Now, there is no conflict. However, there are repeated urges to tilt forward. According to the rules, the tilting forward behaviour will be enhanced from the starting effort level of 1 to the enhanced effort level of 2, and the robot will tilt forward for about 20°—and at the same time left, for about 10°, as required by the “agreeableness” trait node. Why two different personality traits lead to the same urge? Because they have separate meanings. An extroverted forward tilt can be a spontaneous expression of curiosity or attention, whereas a conscientious forward tilt can be a deliberate cue to encourage the interlocutor to elaborate further on the topic in order to acquire more information.

**TABLE 2:** Behavioural mappings.

Broad Traits	Cognitive States	Behaviour^a^	Starting Effort Levels
Extraversion	agreeing	nodding (+)	1
	disagreeing	head shaking (+)	1
	engaged	tilting forward (+)	1
	interested	tilting forward (+)	1
	uncertain	head shaking (+)	1
	annoyed	eye-rolling (+)	1
	annoyed	tilting backward (+)	1
	default	gaze aversion (-)	1
	default	show dominance (+)	-
Agreeableness	agreeing	nodding (+)	1
	agreeing	smiling (+)	1
	disagreeing	smiling (+)	1
	engaged	nodding (+)	1
	engaged	smiling (+)	1
	interested	tilting to the left (+)	1
	interested	smiling (+)	1
	uncertain	tilting to the right (+)	1
	annoyed	nodding (+)	1
	default	polite nodding (+)	1
	default	smiling (+)	1
Conscientiousness	agreeing	nodding (+)	1
	disagreeing	do nothing	-
	engaged	tilting forward (+)	1
	interested	tilting forward (+)	1
	uncertain	tilting forward (+)	1
	annoyed	do nothing	-
	default	do nothing	-

a Behaviour of which the tendency is correlated with the trait level is marked with (+), and that of which the tendency is reversely correlated with the trait level (-).

**TABLE 3:** Classes of behaviour and the levels of effort.

Behaviour	Description	Effort Levels
Nodding	a slight dip	1
	a nod	2
	an up-and-down nod	3
	head bobbing	4
Polite nodding	a slight dip at “polite nodding points”	1
Head shaking	a shake of the head	1
Tilting forward	10°	1
	20°	2
Tilting backward	10°	1
	20°	2
Tilting to the left	10°	1
	20°	2
Tilting to the right	10°	1
	20°	2
Show dominance	a slight ( <10 degrees) head forward tilt offset to show dominance	-
Gaze aversion	extraversion-modulated gaze aversion (lasts about a second)	1
Eye-rolling	eye actuators to up maximum, rolling left to right, and then return to the default position	1
Smiling	pulling the corners of the mouth for 3 s	1
Do nothing	no actuator input	-

**TABLE 4:** Behavioural conflicts.

Conflicting Pairs of Behaviour	
Nodding	head shaking
Nodding	tilting backward
Nodding	tilting to the left
Nodding	tilting to the right
Head shaking	tilting forward
Head shaking	tilting backward
Head shaking	tilting to the left
Head shaking	tilting to the right
Smiling	eye-rolling
Nodding	eye-rolling
Gaze aversion	eye-rolling

##### 5.3.1.1 Eye Movement

Of the known eye behaviour, the correlations between gaze aversion and extraversion were the best understood. Therefore, we included gaze aversion in the mapping function of extraversion trait node. The rate of gaze aversion was correlated with introversion; the probability of generating an urge for aversion was the complement of the probability for extraversion urges. For example, if the trait level of extraversion is 11 out of 100. The probability of an urge for gaze aversion is 0.89. The direction of gaze aversion followed a bivariate normal distribution as introduced in our previous work (Luo et al., 2019). The difference was that we had no human archetypes to learn from this time since our robot personalities were not based on humans. Therefore, we had had the recourse to the results from a recent study that reported that extroverts were more inclined to avert to right up, and introverts left down (Durupinar, 2020). In the prototype model, the trait level of extraversion was also the probability for gaze aversion to be right up, and introversion, left down. We also included eye-rolling to increase the variety of eye behaviour, though due to the limitations of our robot, their eye-rolling was more like a “maximum-effort” gaze aversion. In addition to what is listed in Table 2, the robot was also programmed with random micro-movements like humans' saccades but much slower. It had nothing to do with the robot’s personality and was active all the time.

##### 5.3.1.2 Head Movement

As for head movement, the prototype model handled the following behaviour: nodding (four levels of effort); tilting forward or backward, to the left or right (two levels each), and shaking (one level). In addition, we included a “nodding as an indication of paying attention” or “polite nodding” as a “once on always on” behaviour. When it was on, the robot would offer a slight dip (same as the effort level-1 nodding) at every “polite nodding point”. There was also a “show dominance” behaviour implemented based on the discovery of Witkower and Tracy (2019), since dominance is related to extraversion. However, the tested robot had no eyebrows and the offset was small, and hence it was likely that the effect of showing dominance did not manifest.

##### 5.3.1.3 Facial Expression

Facial expressions require delicate actuators that may have no other use than making facial expressions and are hence a “luxury”. Our robot, albeit designed as a life-size human-robot interaction testbed, could only manage a very rudimentary smile by an actuator that fixedly pulled their cheeks. Due to the limitation of the robot, the smile could not be meaningfully enhanced. Therefore, the smiling behaviour consisted of only one effort level. Considering the significance of smiling behaviour, future humanoid robots, no matter how “barebones” they are, may be engineered with the basic ability to smile or express a similar token, which was why we included this behaviour.

##### 5.3.1.4 Commitment Function

As for the commitment function, since our dummy cognitive system had no understanding of how the world worked and hence could not produce meaningful conditional probabilities to inform the personality model whether certain behaviour worked against a goal, we just made it into an “equal conditional probabilities” dummy that would always give in to an urge.

#### 5.3.2 Robot

Despite the cover story, there was only one robot that played all robot personalities, not several robots of identical appearances. The robot was a life-size yet “barebones” humanoid (Figure 3). Their eyes had two degrees of freedom and neck three. The robot also had an actuator for producing smiles, which was one of the few facial expressions they could manage. The robot’s pneumatic actuators had a response time of about 200 ms, which was close to the average reaction time of humans. The robot was controlled by dedicated proprietary software. The software received actuator commands sent by our prototype actuator controller that worked in conjunction with our prototype model, which was implemented in C++. The actuator controller simply mapped instances of behaviour to the pre-recorded actuator sequences.

### 5.4 Participants

The participants included two members from our lab as the voice actors for HUMAN and ROBOT in the scenario; the 20 participants who annotated our dialogue and assessed the personality expressed by the voice tracks; the 60 participants who acted as the observers in the two tests, 30 each. The 80 outsider participants were all workers on Amazon Mechanical Turk recruited with the qualifications that they must have 5,000 or more approvals and a 90 per cent or above approval rate.

### 5.5 Test 1: Four Robot Personalities

Robot Personalities 1 to 4 joined Test 1. 30 participants completed our survey.

We made pair-wise comparisons by Euclidean distances and cosine similarity since a personality space is typically a Euclidean space and personalities can be represented as Euclidean vectors in the personality space. Each personality as observed by an observer was a 3-dimensional vector. Each observer made eight observations corresponding to the eight videos, which led to 82=28 pairs. There were 30 observers and hence each pair corresponds to 30 Euclidean distances and 30 cosine similarity values. The means of the results of the two metrics are recorded in Table 5. The observers could distinguish all pairs, not only two different personalities but the same personality in two videos as well, as demonstrated by pairs RP-1a and RP-1b (*M* = 1.8, *SD* = 1.3), RP-2a and RP-2b (*M* = 1.8, *SD* = 0.9), RP-3a and RP-3b (*M* = 2.2, *SD* = 1.3), RP-4a and RP-4b (*M* = 1.8, *SD* = 0.9), implying that the observers were unable to identify the personalities because they failed to when the same personalities appeared for the second time. Table 6 shows the distribution of participants as per the numbers of personalities reported. Interestingly, 16 out of the 30 participants reported that they had perceived 5 or more personalities in the 8 videos, whereas the correct number was 4. Combining the results in Tables 5 and 6, we inferred that many subjects might have taken the same robot personalities that behaved differently in two videos for two robot personalities, which is a case nearly impossible with humans since no two humans look identical—even twins have subtle differences. The results imply that when there are no visual cues available for distinguishing multiple individuals, observers may become over sensitive to their behaviour. As for the cosine similarity, the means of all pairs demonstrated high similarity, suggesting that the perceived personalities were all similarly oriented as vectors in the personality space, implying that the Euclidean distances were caused by the differences in the trait levels on all dimensions.

**TABLE 5:** Test 1: Pair-wise personality comparisons.

Pairs^a^ ^,^ ^b^	Mean Euclidean Distances	Mean Cosine Similarity
RP-1a and RP-1b	*M* = 1.8, *SD* = 1.3	*M* = 0.97, *SD* = 0.04
RP-1a and RP-2a	*M* = 1.9, *SD* = 1.2	*M* = 0.98, *SD* = 0.03
RP-1a and RP-2b	*M* = 1.8, *SD* = 1.1	*M* = 0.98, *SD* = 0.03
RP-1a and RP-3a	*M* = 1.9, *SD* = 1.2	*M* = 0.98, *SD* = 0.03
RP-1a and RP-3b	*M* = 2.1, *SD* = 1.5	*M* = 0.97, *SD* = 0.04
RP-1a and RP-4a	*M* = 1.6, *SD* = 0.9	*M* = 0.99, *SD* = 0.01
RP-1a and RP-4b	*M* = 2.0, *SD* = 1.2	*M* = 0.98, *SD* = 0.02
RP-1b and RP-2a	*M* = 1.6, *SD* = 0.7	*M* = 0.98, *SD* = 0.02
RP-1b and RP-2b	*M* = 1.6, *SD* = 1.1	*M* = 0.98, *SD* = 0.03
RP-1b and RP-3a	*M* = 1.9, *SD* = 1.3	*M* = 0.97, *SD* = 0.05
RP-1b and RP-3b	*M* = 1.9, *SD* = 1.2	*M* = 0.98, *SD* = 0.03
RP-1b and RP-4a	*M* = 1.8, *SD* = 1.2	*M* = 0.98, *SD* = 0.03
RP-1b and RP-4b	*M* = 2.0, *SD* = 1.3	*M* = 0.97, *SD* = 0.05
RP-2a and RP-2b	*M* = 1.8, *SD* = 0.9	*M* = 0.98, *SD* = 0.02
RP-2a and RP-3a	*M* = 1.8, *SD* = 1.2	*M* = 0.98, *SD* = 0.04
RP-2a and RP-3b	*M* = 1.9, *SD* = 1.1	*M* = 0.97, *SD* = 0.03
RP-2a and RP-4a	*M* = 1.7, *SD* = 0.9	*M* = 0.98, *SD* = 0.03
RP-2a and RP-4b	*M* = 1.7, *SD* = 1.4	*M* = 0.97, *SD* = 0.05
RP-2b and RP-3a	*M* = 1.7, *SD* = 0.9	*M* = 0.98, *SD* = 0.02
RP-2b and RP-3b	*M* = 2.0, *SD* = 1.3	*M* = 0.97, *SD* = 0.03
RP-2b and RP-4a	*M* = 1.7, *SD* = 1.0	*M* = 0.98, *SD* = 0.03
RP-2b and RP-4b	*M* = 2.0, *SD* = 1.4	*M* = 0.97, *SD* = 0.03
RP-3a and RP-3b	*M* = 2.2, *SD* = 1.3	*M* = 0.97, *SD* = 0.04
RP-3a and RP-4a	*M* = 1.7, *SD* = 1.1	*M* = 0.98, *SD* = 0.03
RP-3a and RP-4b	*M* = 2.1, *SD* = 1.4	*M* = 0.97, *SD* = 0.04
RP-3b and RP-4a	*M* = 1.8, *SD* = 1.2	*M* = 0.97, *SD* = 0.04
RP-3b and RP-4b	*M* = 1.9, *SD* = 1.4	*M* = 0.97, *SD* = 0.05
RP-4a and RP-4b	*M* = 1.8, *SD* = 0.9	*M* = 0.98, *SD* = 0.03

a RP, is short for “Robot Personality”.

b Letters a and b mark two videos of the same personality.

**TABLE 6:** Test 1: The distribution of participants as per the reported numbers of personalities.

Reported Numbers of Personalities	Numbers of Participants Who Thus Reported
1	3
2	1
3	6
4	4
5	8
6	1
7	2
8	5

**FIGURE 5:**
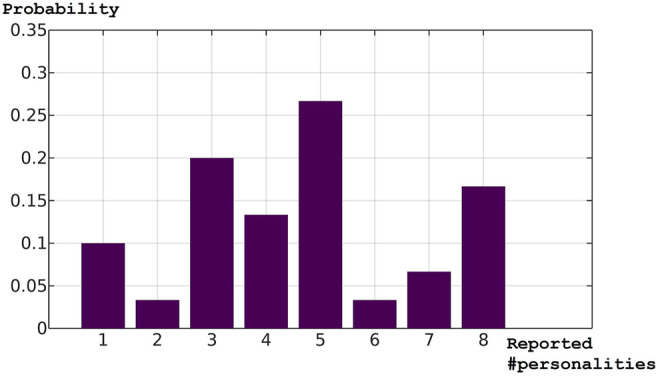
Test 1: The probability mass function of the reported numbers of personalities.

Figure 5 shows the probability mass function plotted from the distribution in Table 6. The shape is close to neither a normal nor uniform distribution; there are two “dips” at two and six to seven respectively. The highest likelihood is to report five personalities, followed by three and eight. The distribution indicates that there is a 66 per cent chance that an observer will report 4 or more robot personalities, and the chance for the correct number is estimated to be 13 per cent.

### 5.6 Test 2: Eight Robot Personalities

We followed the same procedure as in Test 1. Only that, this time, each of the eight videos featured a unique personality. The 30 participants were different from those who participated in Test 1—a between-subjects design—so that there was no inference from the previous results. The eight robot personalities who joined Test 2 were Robot Personalities 5 to 12.

**FIGURE 6:**
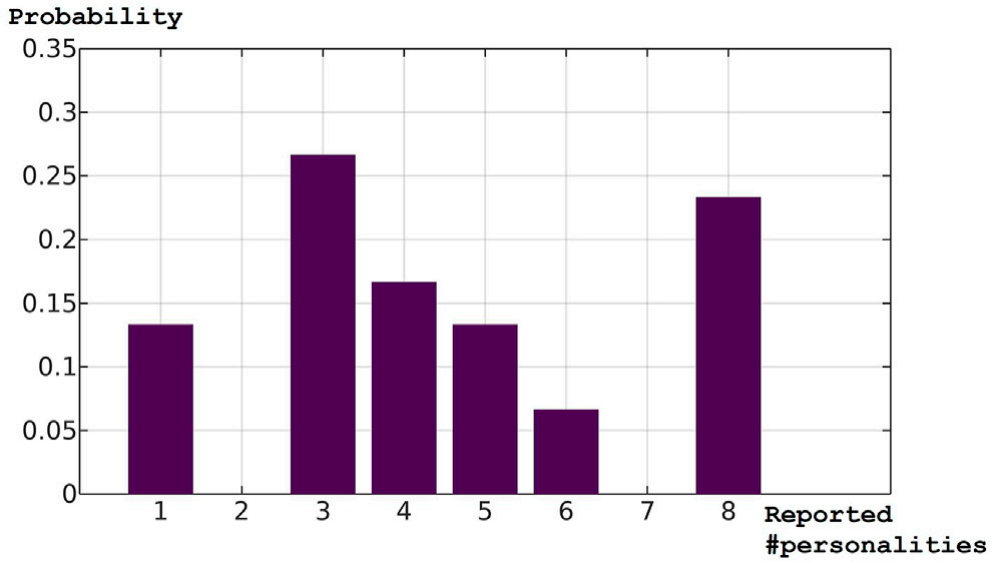
Test 2: The probability mass function of the reported numbers of personalities.

The mean Euclidean distances and cosine similarity values were recorded in Table 7, the distribution of participants as per the numbers of personalities reported in Table 8, and the corresponding probability mass function in Figure 6. As before, the shape of the function was close to neither a normal nor uniform distribution. Compared with Figure 5, the distribution showed a trend towards a “bifurcation” of opinions, as more people had reported either three or eight robot personalities and the middle was dented. The distribution indicates that there is a 60 per cent chance that an observer will report 4 or more robot personalities, and the chance for the correct number is estimated to be 23 per cent.

**TABLE 7:** Test 2: Pair-wise personality comparisons.

Pairs^a^	Mean Euclidean Distances	Mean Cosine Similarity
RP-5 and RP-6	*M* = 2.0, *SD* = 1.3	*M* = 0.98, *SD* = 0.03
RP-5 and RP-7	*M* = 1.7, *SD* = 1.3	*M* = 0.98, *SD* = 0.03
RP-5 and RP-8	*M* = 1.8, *SD* = 1.2	*M* = 0.98, *SD* = 0.02
RP-5 and RP-9	*M* = 1.8, *SD* = 1.2	*M* = 0.98, *SD* = 0.03
RP-5 and RP-10	*M* = 1.8, *SD* = 1.3	*M* = 0.98, *SD* = 0.02
RP-5 and RP-11	*M* = 1.5, *SD* = 1.1	*M* = 0.99, *SD* = 0.02
RP-5 and RP-12	*M* = 1.6, *SD* = 1.2	*M* = 0.99, *SD* = 0.02
RP-6 and RP-7	*M* = 1.5, *SD* = 1.2	*M* = 0.99, *SD* = 0.02
RP-6 and RP-8	*M* = 1.6, *SD* = 1.2	*M* = 0.99, *SD* = 0.01
RP-6 and RP-9	*M* = 1.9, *SD* = 1.5	*M* = 0.98, *SD* = 0.02
RP-6 and RP-10	*M* = 1.4, *SD* = 1.0	*M* = 0.98, *SD* = 0.02
RP-6 and RP-11	*M* = 1.7, *SD* = 1.3	*M* = 0.98, *SD* = 0.03
RP-6 and RP-12	*M* = 1.7, *SD* = 1.1	*M* = 0.98, *SD* = 0.02
RP-7 and RP-8	*M* = 1.4, *SD* = 1.0	*M* = 0.99, *SD* = 0.01
RP-7 and RP-9	*M* = 1.7, *SD* = 1.6	*M* = 0.98, *SD* = 0.02
RP-7 and RP-10	*M* = 1.7, *SD* = 1.3	*M* = 0.98, *SD* = 0.02
RP-7 and RP-11	*M* = 1.6, *SD* = 1.2	*M* = 0.98, *SD* = 0.02
RP-7 and RP-12	*M* = 1.4, *SD* = 1.1	*M* = 0.99, *SD* = 0.02
RP-8 and RP-9	*M* = 1.6, *SD* = 1.2	*M* = 0.99, *SD* = 0.02
RP-8 and RP-10	*M* = 1.7, *SD* = 1.3	*M* = 0.98, *SD* = 0.03
RP-8 and RP-11	*M* = 1.8, *SD* = 1.4	*M* = 0.98, *SD* = 0.02
RP-8 and RP-12	*M* = 1.3, *SD* = 0.9	*M* = 0.99, *SD* = 0.02
RP-9 and RP-10	*M* = 2.1, *SD* = 1.6	*M* = 0.98, *SD* = 0.04
RP-9 and RP-11	*M* = 1.9, *SD* = 1.7	*M* = 0.98, *SD* = 0.04
RP-9 and RP-12	*M* = 1.5, *SD* = 1.2	*M* = 0.98, *SD* = 0.02
RP-10 and RP-11	*M* = 1.9, *SD* = 1.4	*M* = 0.98, *SD* = 0.03
RP-10 and RP-12	*M* = 1.7, *SD* = 1.2	*M* = 0.99, *SD* = 0.02
RP-11 and RP-12	*M* = 1.6, *SD* = 1.4	*M* = 0.98, *SD* = 0.03

a RP, is short for “Robot Personality”.

**TABLE 8:** Test 2: The distribution of participants as per the reported numbers of personalities.

Reported Numbers of Personalities	Numbers of Participants Who Thus Reported
1	4
2	0
3	8
4	5
5	4
6	2
7	0
8	7

The mean Euclidean distances from Test 2 appeared to be smaller. To test the hypothesis that the mean Euclidean distances from Test 2 were smaller than those in Test 1, we applied Welch’s test and discovered a significant difference, *t* (49.29) = 3.72, *p* = 0.0005, and Cohen’s *d* = 1.2: the robot personality pairs in Test 2 were perceived as more similar (*M* = 1.67, *SD* = 0.21) among one another compared with those in Test 1 (*M* = 1.85, *SD* = 0.15), which reflected the configurations of Robot Personalities 5 to 12, which were more evenly distributed than Robot Personalities 1 to 4. This might have caused the trend towards the “bifurcation” of opinions as observed in Figure 6; for it is possible that, observing a larger colony of robot personalities more smoothly distributed in the personality space, the people sensitive to their differences may perceive them as more robot personalities with smaller inter-individual differences, whereas the people less sensitive to subtle differences in similar personalities may perceive them as fewer robot personalities.

### 5.7 Summary of the Results

More than half of the observers in Test 1 reported more than four robot personalities after repeated exposure to each of the four, and there were confusions as to how many personalities were shown to them and whether those in two videos were one. About 60 per cent of the observers in Test 2 observed 4 or more robot personalities and 30 per cent, 6 or more, given that the actual colony size was 8.

Based on the results, we would like to proffer two inferences: 1) when there lack visual cues to distinguish different robot personalities whose appearances are identical, people may become more sensitive to their behaviour in order to distinguish them and report more personalities than there actually are under enough exposure; and 2) for a colony of robot personalities whose appearances are all identical, if their personalities are more similarly configured using our model, they may be perceived as either more or fewer personalities than a colony of which the members are less similar due to that not all people are equally sensitive to subtle differences in behaviour. Whether these two inferences are correct explanations of what we have observed and how humans perceive personalities in physically identical agents require further investigation. However, it is unlikely that real users of robots will be subjected to such guessing games. They will know how many robots are working with them. It is more important that they can distinguish and identify the robots by their personalities. Both tests have shown that the former is possible; Test 1 showed that the latter might be very difficult.

## 6 Conclusion

### 6.1 Discussions

Given the results, what can be concluded with certainty is that a generative personality model as proposed can make physically identical robots appear as multiple personalities: the effective colony capacity as demonstrated ranged from four to eight according to most observers, which is good news because engineering mass-produced robots into significant quantities of robot personalities for practical applications is the number one challenge of robot personalities engineering. However, the results have revealed a critical issue to address.

There was clearly confusion as to how many personalities there actually were in the two tests. More than half of the subjects in Test 1 reported more than four, the actual number, and they could not tell if the characters in two videos were in fact the same personality, as demonstrated by Table 5. The inability to identify multiple robots is a potential issue for environments where there are multiple robot personalities with highly similar or even identical appearances, as the human brain is probably not tuned to handle such situations. There is still much we do not understand about how humans perceive personalities in physically identical agents; however, even mass-produced robots do not have to look exactly the same. Considering that it is at least practical to implement simple visual cues, such as symbols and liveries, and accessories and simple clothing, anything safe and also cheaper than customised appearances, we recommend combining personality expressions with visual cues to distinguish and identify robots and leverage other benefits of robot personalities that are similar since not all people are sensitive to subtle differences in personality expressions. There can be a multitude of future studies on the types of visual cues (e.g., colours or patterns of symbols or liveries) and their effectiveness. For every type of simple visual cues, we can test these hypotheses: the visual cues improve the accuracy of 1) identification and 2) differentiation of robot personalities engineered out of the same model of robot. We can also investigate whether simple visual cues help enhance the sensitivity to subtle differences in personality expressions for those who are less sensitive (e.g., those who reported one, two, or three robot personalities in our experiment). Another crucial investigation should be on the impact of visual cues to the impressions of robot personalities, such as how do visual cues with meanings and cultural significance change the impression. Would the same robot personality be perceived differently when it is painted with flowers as compared with animals? Would the species of animals make a difference too? Such as a dire wolf compared with a golden lion on the same robot. Conversely, we can also study whether personalities improve the effectiveness of visual cues, such as in a study with the same group of robots, each with a unique name and livery, in two conditions, one of which features personalities and one does not, to see if the subjects can better match the names to the liveries with the help of the robots’ personalities.

Due to the limitations of our robot and other limitations such as our limited understanding of the relationships between personality broad traits and behaviour and the limitation of the subject experiment (exposure offered by short videos is highly limited), we were unable to explore the full potential of the proposed model. We have likely underestimated the effective colony capacity by a large margin. Nevertheless, the current results are sufficient as the basis to continue developing the model. The effective colony capacity should increase as robots and our understanding of personality improve.

Although we describe the proposed model as “goal-shaped,” we have not yet tested the shaping mechanisms as described by Heuristic B. Instead, we tested 12 personalities that were already “shaped” as part of the settings for the experiment. Therefore, future work should cover personality development and “shaping by goals” mechanisms by assigning different robots with different goals that would shape their personalities differently overtime. Only then can we acquire a complete assessment of the proposed model. To do that, we will need some robots that can handle a variety of jobs. Hopefully, that day can arrive sooner.

Another research area essential to constructing practical models is the study of behavioural mappings, which can be divided by modes or modalities. We were not satisfied with the mapping functions in Table 2, which were mostly based on our intuitions and lacked empirical support. The mappings of the conscientiousness node lacked variation of behaviour due to our insufficient understanding of how a conscientious person would behave in the test scenario. The mappings in the other two trait nodes might not be accurate enough for practical applications. An important task of robot personalities engineering is to figure out accurate behavioural mappings. Our experiment only featured mapping functions for non-verbal behaviour on the head, of which the limitation is evident in expressing multiple personalities without confusion. Future work should cover speech and gestures despite the challenges. As for speech, the mapping functions would be much more complicated then “a cognitive state to a behaviour” mapping. What might be practical for now is mapping cognitive states to emotions in speech with adjustable strength. That said, the ultimate goal should be characteristic speech: enabling robots to say lines full of character as how playwrights breath life into their characters. As for gestures, other than the usually problems of what cognitive states should be mapped to what gestures to express what personality traits, a major challenge is safety, such as collision avoidance and maintaining balance while gesturing, which are easy for a human to do but tricky and risky for a 200-kg life-size humanoid robot that falls with a terrifying thud. However, the research could start with smaller humanoid robots.

Of the four criteria for assessing a generative personality model, we covered colony capacity in this study. Trait capacity is less of an issue to the expandable architecture of our model, which has no limit on the number of trait nodes. Still, a large number of trait nodes can slow down the computation, which is a common issue of large-scale graphical models. Future work should cover consistency and fidelity of the model. Only a model that can present multiple personalities with fidelity and consistency is suitable for practical applications. However, these two criteria depend not on the high-level designs presented in this paper, but the build of a particular model, which further depends on the type of robot, its usage, and the corresponding behaviour it handles. Therefore, more limited scopes apply. To achieve high fidelity and consistency, we need dedicated optimisation methods, which can be another area of future research. Our previous work provides some preliminary discussions on this topic (Luo et al., 2022).

Our experiment covered cases with four or eight robot personalities. We started with four since we had aimed for engineering robot personalities in significant quantities. However, the reader might be curious as to what would happen if there were only two personalities. While it is easy to assume that two robot personalities would be easier to distinguish and identify after enough exposure, as how parents of twins tell their children apart, the cues and circumstances involved are worth studying using robots so that the results could help engineer robot personalities that can be more easily distinguished and identified. Here, we can employ human twins as archetypes and let the same robot imitate them. Because it is possible to switch on and off at will modes of personality expressions on a robot, a great advantage robots have over humans, we will be able to conduct better controlled experiments to identify essential cues for distinguishing and identifying robot personalities with similar appearances.

### 6.2 Conclusions

In this study, we proposed a goal-shaped generative personality model for the personality AI towards which we had been working and tested the effective colony capacity of a prototype model for expressing personalities *via* the non-verbal behaviour on the head of a humanoid robot. The prototype modelled three traits of the five-factor model, extraversion, agreeableness, and conscientiousness, under design constraints that agreeableness and conscientiousness trait levels must be higher than half of the maximum. Our test results indicated that most observers could observe in a colony of physically identical robots more than four robot personalities when the colony size was equal to or larger than four, but with substantial confusion about the exact numbers. And they could not recognised the same personalities when they appeared for the second time, implying an issue in identification. The tests were done under many constraints such as imperfect understanding of the relationships between traits and behaviour, limited exposure, the limitations of the robot, and so on. With future improvements on behavioural mappings and personality development and shaping mechanisms, the model has the potential to present even more personalities more accurately. For the limited questions we answered, a host of new problems have emerged, which we could only shovel to the discussion section, swelling it into a monstrous mass of uncertainty dwarfing the little piece of knowledge we could offer. We would like to consider this both a limitation and strength of this work. We hope the reader could find in that mass of uncertainty something interesting to pursue.

### 6.3 Final Remarks

Those robots who are going to work side by side with humans will be limited at least for a while. But oftentimes, limitations lead to infinite possibilities.

## Data Availability

The raw data supporting the conclusion of this article will be made available by the authors, without undue reservation.

## Footnotes

1 It may be called a “modality” in another context. Here it is not because the nuances are different. A mode of expression is not necessarily *how* a robot expresses personality; the robot, albeit equipped with modes, may not express personality at all, or it may *happen* to express personality (spontaneously).

2 The actuators do not necessarily need to actuate anthropomorphic features to express personality, as demonstrated by BB-8, a robot spacecraft mechanic in *Star Wars: The Force Awakens*, who made a “thumbs-up” gesture using a welding torch.

3 The personality model in Figure 1 is an example. A personality construct based on the proposed model does not necessarily have five trait nodes, six goal nodes, and a goal node or more connected to each trait node.

4 Two sample videos can be found *via* https://youtu.be/1cwJiQKBWIQ and https://youtu.be/XgDkpSVtIZ4.
